# Stable Strontium Distribution and Effects on Plants

**DOI:** 10.3390/ijms27209235

**Published:** 2026-10-17

**Authors:** Ilya V. Seregin, Anna D. Kozhevnikova

**Affiliations:** https://ror.org/03hg8g849 K.A. Timiryazev Institute of Plant Physiology, https://ror.org/05qrfxd25 Russian Academy of Sciences, Botanicheskaya St., 35, Moscow 127276, Russia

**Keywords:** enzyme activity, mineral nutrition, photosynthesis, plant growth and development, seed germination, strontium, strontium uptake, transport and distribution

## Abstract

Strontium (Sr) is a wide-spread alkaline earth metal that is similar to calcium (Ca) in its chemical properties, although it does not play the physiological role inherent to the latter. At low doses, Sr can stimulate plant growth and development. However, at elevated levels in the environment, it has a negative effect on many processes occurring in plant cells, leading to growth inhibition and reduced productivity. The majority of studies have been focused on the effects of radioactive Sr isotopes which produce additional toxic effects due to ionizing radiation, but the effects of stable Sr have not received enough attention. The current scoping review summarizes the data on the uptake mechanisms and transport pathways of stable Sr and examines the characteristic patterns of its distribution in plant tissues and organs. Available data on the effects of stable Sr on enzyme activity, mineral nutrition, photosynthesis, plant growth, and morphogenesis are analyzed. Understanding the transport pathways of Sr, as well as the factors determining its dual effects on plant growth and development, underlies the development of approaches used in biofortification and phytoremediation technologies. The review may be useful for researchers working on plant mineral nutrition, metal stress, and phytoremediation.

## 1. Introduction

Due to increased industrial activity, potentially toxic elements have recently received considerable attention, as they continue to cause global concern due to their danger to the environment [1]. These include elements essential for plants, such as copper (Cu), iron (Fe), manganese (Mn), molybdenum (Mo), nickel (Ni), and zinc (Zn), as well as highly toxic and nonessential ones, such as lead (Pb), cadmium (Cd), chromium (Cr), and mercury (Hg). At supra-optimal concentrations, even essential elements can directly or indirectly exert multiple toxic effects on a variety of physiological processes [2,3,4,5], which leads to inhibition of plant growth and a decrease in their productivity [6]. Strontium (Sr) is a Group IIA alkaline-earth metal and an analogue of calcium (Ca), which belongs to the same group of chemical elements. They have quite similar physical properties such as ionic radius and charge [7]. Strontium is often used as a tracer for Ca transport in plants [8,9,10,11,12], but cannot totally mimic Ca [13], as it is a non-essential element and does not play the physiological role inherent to Ca [7].

The physicochemical properties of ions largely determine both the mechanisms of their transport and their ability to interact with various molecules in plant cells. The radius of the Sr ion is 0.113 nm. The interactions of ions with water molecules and biological ligands are determined by the values of the ionic potential and the Misono softness parameter. The ionic potential (φ), which is the ratio of the ionic charge (Z) to the ionic radius (r), determines the strength of the interaction of metal ions with neighboring water molecules in dilute aqueous solutions. If the ionic potential is below 30, metal cations stay solvated. The ionic potential of Sr ions is 17.7 nm^−1^ [14], indicating their solvation by water molecules. The Misono softness parameter characterizes the tendency of a metal cation to polarize and evaluates how easily it forms covalent bonds, based on its ionic radius and ionization potential. Based on its values, metal ions are classified into hard and soft Lewis acids. Hard Lewis acids are characterized by high positive charge density, low polarizability, and form complexes via electrostatic interactions, whereas soft Lewis acids have low positive charge density, high polarizability, and form stable complexes via covalent interactions. The Misono softness parameter determines the affinity of ions for ligands. Hard Lewis acids have a higher affinity for O-containing ligands, while soft Lewis acids have a higher affinity for S-ligands. The Misono softness parameter of Sr ions is 0.202 nm [14], indicating their preferential interaction with O-containing ligands.

Naturally occurring Sr is abundant in the Earth’s crust and, ranking 15th, occurs as a mixture of four stable isotopes (^84^Sr, ^86^Sr, ^87^Sr, ^88^Sr), primarily ^88^Sr (~83%). Strontium is found as carbonate (SrCO_3_) and sulfate (SrSO_4_) in such minerals as strontianite and celestite, respectively [7,15]. Due to the Chernobyl (in 1986) and Fukushima (in 2011) nuclear disasters, a lot of attention has recently been paid to studying the effects of radiostrontium on plants accumulating and providing Sr into food chains [7,16]. Much fewer studies have examined the effects of stable, non-radioactive Sr on plants, although this element often enters the environment in significant quantities, in particular due to weathering of minerals containing Sr [17] and its extensive use in fireworks industries [18]. Concentrations of stable Sr usually range from 5 to 3100 mg kg^−1^ in soil, with the highest levels in heavy loamy soils, and from 0.5 to 8000 µg L^−1^ in water. The main sources of Sr pollution are coal combustion, sulfur mining and processing, and extensive applying of phosphorus (P) fertilizers due to elevated amounts of Sr in phosphorites (up to about 2000 mg kg^−1^ Sr) [19]. Stable and radioactive isotopes of Sr exhibit identical chemical properties. Therefore, in experimental studies, stable Sr is often used instead of radioactive Sr in order to reduce the risk of radiation exposure and avoid the problems related to processing of experimental waste [20,21,22]. Unravelling the mechanisms of the toxic effects of stable Sr will help to distinguish between the effects induced by the Sr element per se from ones resulting from radioactivity. At the same time, Sr at low doses can stimulate plant growth [22,23,24,25,26,27,28]. In humans, Sr also plays a dual role. Low doses of Sr may stimulate osteogenesis, whereas high doses pose significant health risks [29,30]. The daily intake of Sr for humans is approximately 2.0 mg Sr/day/person, with plant foods containing more Sr than animal foods [26]. Therefore, studying its effects on plants is not only of fundamental but also of great practical importance.

Despite the fact that the study of Sr uptake, transport, and effects on plants began in the first half of the 20th century, our knowledge on this topic is still far from being complete. The majority of studies and reviews have been focused on the effects of radioactive Sr isotopes, but the effects of stable Sr have not received enough attention. A recent review by Burger and Lichtscheidl [7] provided a comparative analysis of plant interaction with stable and radioactive Sr isotopes, with a specific focus on the mechanisms of Sr uptake, the sources and toxic effects of radiostrontium, Sr distribution in plants and remediation of Sr-polluted soil and water. However, a significant amount of new studies has appeared since 2019, especially on the effects of stable Sr on plant physiological processes. To perform our scoping review, we conducted a systematic literature search in PubMed and Google Scholar databases using the search terms ‘strontium in plants’, ‘strontium toxic effects on plants’, ‘strontium uptake’, ‘strontium transport in plants’, ‘strontium localization in plants’, ‘strontium accumulation in plants’, ‘strontium effect on plant growth’, ‘strontium effect on plant development’, ‘strontium effect on respiration’, ‘strontium effect on photosynthesis’, ‘strontium effect on mineral nutrition’, etc. The search was limited to English-language articles, and covered the full date range available up to October 2026. We manually screened these articles, excluded ones dedicated solely to radiostrontium and discharged studies not relevant to the topic of the current review or bearing low scientific soundness due to the lack of statistical analysis of the data or to the use of only close to lethal Sr concentrations. We have comprehensively summarized the data on the effects of stable Sr on various physiological processes in plants. Only scarce data on the effects of stable Sr on plant respiration and water balance have been found and will be reported. The major focus will be on the effects of stable Sr on enzyme activity, mineral nutrition, photosynthesis, plant growth, and morphogenesis. The transcriptomic and miRNA-related responses as well as other recent mechanistic findings will also be discussed. The modern concepts regarding the absorption, transport, accumulation and distribution of Sr in plant tissues and organs will be analyzed.

## 2. Strontium Uptake and Transport in Plants

Strontium enters plants primarily through the root system from the soil, where it is unevenly distributed [31]. As young roots readily absorb Sr, it was used as a tracer to detect root activity in *Dioscorea alata* [32]. In *Zea mays* seedlings, Sr was absorbed both in the root apex and mature regions. The uptake of stable Sr was shown to be a non-metabolic process in the meristem, but was dependent upon metabolism in the vacuolated tissues [33]. The highest rate of Sr uptake was observed in the root elongation zone, with a lower rate in the root hair zone and the lowest rate in the meristem. Most of the absorbed Sr (67–70%) was relatively firmly retained by cells in all root zones [34].

The rate of Sr uptake depends on various environmental factors: the content of macro- and microelements in the soil or solution, pH, aeration, temperature, soil composition and its cation exchange capacity, the morpho-physiological characteristics of plants, as well as soil biological networks built by microbial communities and mycorrhizae [7,35,36,37,38,39,40]. Growing plants under different experimental conditions makes it difficult to compare the results obtained by different researchers.

To date, numerous studies have been conducted on the uptake of radioactive Sr isotopes by plants, while the data on non-radioactive Sr are scarce. An increase in pH in organic soil due to the addition of Ca led to preferential absorption of Ca by plants, whereas the uptake of stable Sr was decreased [7]. Soil amendments with organic matter in some cases alleviated Sr toxic effects on shoot growth of *Brassica rapa* after 80 days of treatment [40], which may be due to a decrease in Sr uptake. Plant treatment with organic nitrogen forms (urea and allantoin) increased Sr content in the shoots of *Hypericum perforatum*, whereas nitrogen deficiency had an opposite effect. At the same time, Sr content in the roots of *Hypericum perforatum* did not change under all treatments compared to the control. Overall, this indicates a positive effect of organic nitrogen forms not only on the absorption of Sr, but also on its root-to-shoot translocation [39].

The uptake of Sr^2+^ by *Zea mays* roots was shown to result from the functioning of at least two saturating (in the range of 0.04–25 mM), and one non-saturating (for Sr^2+^ concentrations up to 100 mM) transport components. Saturating kinetics at relatively low concentrations could suggest the involvement of two transport systems such as carriers or pumps in Sr^2+^ uptake. Linearity of the absorption kinetics for Sr^2+^ at concentrations ranging from 25 to 100 mM pointed to the influx of Sr^2+^ into the root by diffusion at high external Sr levels. The fact that no saturation was found for the concentrations up to 100 mM and the magnitude of the flux might suggest that this Sr flux could be due either to Sr entry into the root by apoplastic diffusion or its entry into the symplast of root cells by diffusion across the plasma membrane, possibly being facilitated via a high-conductance transport system such as cation channels [13]. The involvement of low-affinity transport systems in Sr translocation is evidenced by the correlation between the Sr content and the contents of other elements [41,42].

The molecular mechanisms of Sr transport in plants are insufficiently studied, but it is clear that Ca and potassium (K) transporters are involved in this process [7]. This is indirectly evidenced by a decrease in the contents of these elements in Sr-treated plants, although not at all studied Sr concentrations and not in all organs tested [20,22,25,26,43,44,45,46]. At low Sr levels in the environment, Ca accumulation may not change [28,45]. It is generally accepted that Sr, being an analogue of Ca, can enter the cytoplasm and be transported throughout the plant via non-selective transport pathways for mono- and divalent cations (e.g., Ca^2+^, K^+^, barium (Ba^2+^), magnesium (Mg^2+^), rubidium (Rb^+^)), in particular, via non-selective ion channels [47,48,49]. Strontium entry into the cell through Ca channels, which can be blocked by lanthanum (La^3+^) and gadolinium (Gd^3+^), as well as exit from the cell and transport across the tonoplast via Ca-transporting ATPases have been proposed as possible mechanisms of Sr transport in the unicellular green alga *Erenaosphaera viridis* [50]. The uptake of Ca^2+^ and Sr^2+^ in *Solanum lycopersicum* was significantly inhibited by these blockers, indicating that Ca^2+^ and Sr^2+^ share a part of the channel pathway [11]. Recently, it has been demonstrated that cyclic nucleotide gated channels (CNGCs), which belong to a large family of CNGC proteins, may be involved in Sr transport. Currently, 20 *CNGC* genes have been identified in *Arabidopsis thaliana*, and 16 *CNGC* genes have been proposed to function in *Oryza sativa* [51]. It is possible that AtCNGC1, 3, 9, 11, 12, and 17 may be involved in the transport of Sr [48,52]. Co-expression of *AtCNGC2* and *AtCNGC4* promoted the transport of Sr^2+^, Ca^2+^, Ba^2+^, and K^+^ [53]. In addition to CNGC, Sr can be transported across membranes via depolarization-activated and hyperpolarization-activated Ca^2+^ channels, as well as depolarization-activated outward-rectifying K^+^ channels (KORCs) [48,49,54].

Wood burning, fly ash from coal combustion, and some other processes contribute to the atmospheric input of stable Sr [7]. Therefore, in addition to Sr entry through the root system, under conditions of aerotechnogenic pollution, Sr can enter plants in limited quantities through the leaves, since its redistribution via the phloem, like that of Ca, is generally limited [55,56], although cannot be completely excluded. About 11–17% of Sr entered the fruits of *Malus* × *domestica* from Sr applied directly to the leaves [10]. The possibility of Sr entry through the leaves is also evidenced by an increase in its content in *Oryza sativa* seedlings after foliar spraying [57]. X-ray fluorescence spectroscopy imaging of *Solanum lycopersicum* leaf tissue revealed that foliar-applied Sr was transported towards the leaf margin primarily through the apoplast [12].

## 3. Strontium Distribution in Plant Organs

The distribution of metals in plant tissues and organs can be uneven, which is determined by the heterogeneous localization of transporters, cation channels, and metal-binding ligands [58]. Different plant species can vary significantly in their ability to accumulate Sr [7,31,36,59,60,61,62,63]. Strontium content in marine organisms is generally higher than in terrestrial organisms, and in sea algae, it ranges from 0.026 to 0.14% on a dry weight basis [64]. Among the tested woody species, *Picea abies* accumulated more Sr than *Acer pseudoplatanus* and *Fagus sylvatica* [65]. A comparative analysis of soil-to-plant Sr transfer factors for 23 plant species from the Lillebukt Alkaline Complex at Stjernøy in Northern Norway revealed that Sr content was the lowest in grasses, intermediate in dwarf shrubs, and the highest in herbs. The highest Sr content (2300 mg kg^−1^) was found in a leguminous plant species *Vicia cracca* [31].

Differences in the patterns of Sr accumulation can be observed not only between different species but also between different cultivars of the same species [62,66,67,68]. For example, significant differences in Sr accumulation were shown for the cultivars of *Avena nuda*, *Avena sativa*, *Hordeum vulgare*, and *Triticum aestivum* [62]. The existence of intraspecific differences in plant capacity to accumulate metals can prevent from making an appropriate comparison of the results obtained in different studies, even ones carried out under identical experimental conditions, if the cultivar/variety is not stated.

Strontium content in different plant organs may vary (Table 1). In *Acer pseudoplatanus*, *Fagus sylvatica*, *Picea abies* [65], *Populus alba*, *Populus russkii* [69], *Apium graveolens* [21], *Hypericum perforatum* [39], *Lactuca sativa*, *Raphanus sativus* [20], *Oryza sativa* [57], *Triticum aestivum* and *Triticum durum* [70], the Sr content in the roots was higher than that in the aboveground organs. In *Brassica oleracea*, the Sr content in the roots and outer leaves (which did not compose any cabbage-head) was higher than in the stems and inner leaves (which composed a cabbage-head), and decreased in the presence of Ca [71]. At the same time, in *Amaranthus tricolor* at 0.6–6 mM Sr [27], *Eichhornia crassipes* at 0.3–3 mM Sr [72], *Glycine max* at 0.5–3 mM Sr [24], *Monochoria hastata* at 3 mM Sr [72], *Vicia faba* at 2.84–11.36 mM [45], and also in *Zea mays* at 0.1–10 mM Sr in the growth medium [13], the metal content in the roots was significantly lower than that in the shoots. In *Iris × germanica*, Sr accumulated efficiently in all plant organs, with the highest Sr content found in leaves, followed by roots and rhizomes [73]. The predominant accumulation of Sr in leaves compared to roots and stems was shown for *Brassica napus* at 10–40 mM Sr after 14 and 21 days of exposure [74], as well as for many other species growing either in hydroponics or in soil [59,61,75,76,77,78,79,80].

A more detailed analysis of Sr content in different plant organs also revealed interspecific differences. In *Phragmites australis*, Sr content decreased in the order leaves > roots > rhizomes > stems [85]. In *Avena sativa* and *Sorghum bicolor*, Sr accumulation ranged in the order leaves > roots > stems [66,86], whereas in *Glycine max*, it decreased in the row leaves > stems > roots > seeds. The low Sr content in seeds is quite predictable, since the transfer of non-essential metals to generative organs is usually restricted [25].

The accumulation of Sr in different parts of the root can vary, largely depending on the Ca content in the medium. In the absence of Ca, Sr accumulation was higher in the apical part than in the basal part of the *Zea mays* root. However, Sr content increased over time only in the apex. In contrast, in the presence of Ca, the Sr content in the apical part of the root increased only slightly over time, while more intense Sr accumulation was observed in the basal part of the root. As a result, after just two days of incubation, the Sr content in the basal part of the root was more than five times greater than in the apical one [84].

The Sr accumulation in roots and shoots can change with increasing metal concentration in the medium, which is determined by the ratio of metal uptake and its root-to-shoot translocation. The ratio of total Sr content in the aboveground organs of *Pisum sativum* to its content in the roots increased with relative metal concentration in the solution [87]. The content of Sr in the roots, stems, and leaves of *Brassica napus*, *Brassica oleracea*, *Glycine max*, and *Vicia faba* increased with Sr concentration in the nutrient solution [25,45,71,74], while that in *Avena sativa*—with Sr content in soil [66]. Similar patterns were found in the roots and shoots of *Apium graveolens* [21], *Lactuca sativa*, *Raphanus sativus* [20,22], *Zea mays* [13], as well as the leaves of *Alstonia scholaris* [46], *Brassica rapa* [28] and *Plantago major* [44].

Strontium distribution among plant organs can be significantly dependent on their age. Preferential Sr accumulation in older leaves compared to younger ones was found in *Beta vulgaris* [88] and *Picea abies* [65]. The total Sr content in the aboveground parts of *Oryza sativa* during different developmental stages (tilling stage, panicle formation stage, ripening stage, and harvest time) was dependent on plant weight [89].

In contrast to excluders, which accumulate metals primarily in roots, there are over 800 hyperaccumulator plant species, which, under natural growth conditions, can accumulate significant amounts of metals primarily in shoots without any visible metabolic disturbances. There are certain threshold values for metal/metalloid content in shoots (mg kg^−1^ dry weight), above which species are classified as hyperaccumulators, and these values vary for different metals [90,91]. Hyperaccumulators are characterized by high metal tolerance, which is based on efficient mechanisms of metal sequestration, exclusion, and chelation, aimed at maintaining metal homeostasis and reducing the manifestation of their toxic effects on plants [3]. The hyperaccumulator of Ni, zinc (Zn), and cadmium (Cd) *Noccaea caerulescens* and the hyperaccumulator of Zn and Cd *Arabidopsis halleri* are model species widely used for studying the mechanisms of hyperaccumulation of these metals [58].

Since the Ca content in the leaves of *Arabidopsis halleri* is usually quite high, and Sr, being an analogue of Ca, is transported via the same pathways, a significant accumulation of not only Ca but also of Sr in the aboveground organs of this species was expected. However, unlike *Brassica napus*, *Brassica oleracea* and *Plantago major* [44,71,74], the Sr content in the leaves of *Arabidopsis halleri* reached its maximum at 0.1 and 1 mM Sr, and significantly decreased at higher metal concentrations in the nutrient solution (10 and 100 mM Sr). At the same time, the values of transfer factors for solution-to-plant leaf (on dry weight basis) decreased from 184 at 0.01 mM Sr to 0.01 at 100 mM Sr [82]. When *Alstonia scholaris* was grown hydroponically at 3 mM Sr, the average Sr content in the leaves reached 8705 mg kg^−1^ dry weight, which allowed the authors to classify this species as a hyperaccumulator of Sr [46]. However, without analyzing the Sr content in *Alstonia scholaris* growing in the natural habitat, it is premature to consider this species a hyperaccumulator.

The ability of certain plant species to accumulate significant amounts of Sr in shoots while being tolerant to its excess makes them promising candidates for the development of new methods and approaches in cost-effective and environmentally friendly “green” technologies. Such technologies include phytoremediation, aimed at cleaning or revegetation of contaminated areas [92,93,94], and biofortification, aimed at increasing the level of bioavailable elements in plants [95]. Bioconcentration factor (BCF) and translocation factor (TF) are often indicatively used to assess the phytoremediation potential of a species. The former characterizes plant capacity for metal bioconcentration from soil, and the latter characterizes plant capacity for metal root-to-shoot translocation. However, it is important to note that there are several methods for calculating the BCF and TF values [96]. The higher the translocation factor (greater than 1), the more promising the species is considered to be for phytoextraction aimed at transferring the pollutant to the aboveground organs, which are then utilized. Conversely, the lower these values (lower than 1), the more promising the species is for phytostabilization, which is based on the use of plants to convert metals into a poorly available form, mainly in the plant root system.

The Sr TF, that is, the ratio of Sr content in shoots to that in roots, exceeded 1 for many of the studied plant species, although it varied depending on the Sr concentration in the environment [25,27,46,61,73,77,80,83,86], as well as the duration of treatment [20]. Gender-based differences in Sr translocation were found in *Populus cathayana* and *Salix babylonica* [97]. Species with a significant phytoremediation potential include not only the Zn and Cd hyperaccumulator *Arabidopsis halleri* [82], but also *Amaranthus tricolor* [27], *Plantago major* [44], *Astragalus gummifer*, *Euphorbia macroclada*, *Verbascum cheiranthifolium* [59], *Typha latifolia* [61] and some others [60,61,66]. The Sr phytoremediation efficiency may be enhanced by microbial inoculation and applying nitrogen fertilizers (urea), as was shown for *Sorghum bicolor × sudanense* [98]. Although the Sr hyperaccumulator status of *Alstonia scholaris* remains to be confirmed, it is a good candidate for the use in phytoextraction technology [46]. The aquatic plant *Pontederia crassipes* (formerly *Eichhornia crassipes*) [72] is promising for Sr rhizofiltration, which utilizes plant roots to absorb pollutants from aqueous solutions. *Glycine max* [24] and *Lactuca sativa* [99] have been proposed as promising species for biofortification of Sr-contaminated soils. Further studies of the mechanisms that determine the ability of different plant species to selectively accumulate Sr in roots or shoots will enable the development of new promising methods and approaches for phytostabilization and phytoextraction, respectively.

## 4. Strontium Distribution in Plant Tissues

Data on the main patterns of Sr distribution over root and shoot tissues in various plant species are limited, partly due to the insufficient development of methods for studying metal distribution in living plant tissues. For this purpose, we developed a histochemical method for Sr determination using sodium rhodizonate, which forms a brownish-red complex with Sr [100,101].

Strontium distribution in the tissues of germinating *Zea mays* caryopses was highly uneven. After one day of treatment, large amounts of Sr were detected in the cell walls of the integument cells, the aleurone layer, and several outer layers of the endosperm and scutellum. In the embryo, Sr was found primarily in the cell walls of coleorhiza, coleoptile, radicle, and shoot cells, as well as in the transition zone between the radicle and the shoot [102].

The distribution of Sr in the roots and shoots of *Zea mays* seedlings has been studied in most detail [100,103,104] (Figure 1). Upon entering the root cap cells, Sr was distributed fairly evenly, although its content in the columella cells was usually lower [104]. Since the outer cells of the root cap and the columella cells differ in their ultrastructure and functions [105,106], it can be concluded that the differences in the structure of different root cap cells do not significantly affect Sr distribution therein. The distribution of Sr in the root cap cells can vary significantly depending on the presence of other ions. Thus, in the presence of Ca (3 mM), Sr was detected only in the outer cells of the root cap, which may be due to the competition for common binding sites during their uptake and transport [103,104,107].

Similarly to that in the root cap, Sr distribution in the root meristem of *Zea mays* was quite uniform. Sr accumulation in the cells of the quiescent center and the surrounding cells did not differ significantly [104]. The cells of the quiescent center often have thickened primary cell walls, which, however, practically do not restrict the entry of Sr into these cells. It is plausible that the apoplast of the quiescent center cells is permeable to Sr ions, as it is probably permeable to Ca [108]. In the presence of Ca, Sr was detected only in the outer cell layers of the meristem [104], which may be partly determined by competition for common binding sites during Sr and Ca transport along the apoplast [100,109,110]. A decrease in Sr accumulation in the presence of Ca may be the reason for a decrease in the toxic effect of Sr on root growth [104]. Interestingly, despite Ni accumulation mainly in the protoplasts [103,111,112], upon Ni addition to the medium, Sr accumulation in plant tissues also decreased [113], which indicates possible competition during their transport, probably through cation channels. The study of the combined effects of metals is important in connection with the problem of environmental pollution and the development of various methods and approaches to phytoremediation [92,93,94].

The localization of Sr predominantly in the apoplast of rhizodermal cells in *Zea mays* [100] indicates that Sr transport along the apoplast begins in the rhizodermis (Figure 1). Root cortex occupies a significant cross-sectional area of the root, which can result in the accumulation of large amounts of Sr in it [103]. By binding Sr, the cell walls of the cortical cells can limit its entry into the cytoplasm, which simultaneously serves as an important detoxification mechanism aimed at metal sequestration in a relatively metabolically inactive cellular compartment. Analysis of Sr distribution in plasmolyzed cells revealed that, in addition to cell walls, Sr was also present in the periplasmic space and on the protoplast surface [103], potentially influencing the structure and functioning of the plasma membrane. In mature zone of the *Zea mays* root, the cell walls of the exodermal cells (outer layers of the cortex) are thickened, which, however, does not prevent Sr from entering the underlying cells. This indicates that exodermis is not a barrier for Sr transport [100,103].

Having been transported through the apoplast in the multilayered cortex of the *Zea mays* root, Sr quickly penetrates the endodermal cells, with significant amounts being present in the protoplasts [100]. It can be assumed that Sr is transported through the endodermis along the symplast, as it is typical for Ca ions [47,109,110,114]. After passing through the endodermis along the symplast, Sr is transported across the plasma membrane and enters the apoplast (Figure 1), as evidenced by its localization mainly in the apoplast of pericycle cells [100].

The accumulation of Sr in root cortical cells and central cylinder tissues may depend on its concentration in the growth medium. For example, in *Plantago major* roots, more Sr was found in the central cylinder than in the cortex at 1 mM Sr in the medium, whereas at 100 mM Sr, more metal was accumulated in the cortex [44].

Compared to other metals, which are transported along the root tissues primarily through the apoplast, Sr is highly mobile [100,103]. Its high mobility may be determined by both its relatively low affinity for cell wall material and its ability to be transported through the periplasmic space and cross the endodermal barrier via the symplast, resulting in its rapid entry into the vascular tissues and transport to the shoots.

In shoots, as in roots, Sr is transported primarily along the apoplast. Creger et al. [75] observed Sr accumulation in leaf veins and petioles and, to a lower degree, in mesophyll cells. X-ray microanalysis showed that in *Citrus jambhiri* leaves, Sr accumulated least in bundle sheath and epidermal cells, reaching intermediate levels in spongy mesophyll, while the highest Sr content was found in palisade mesophyll. Strontium moved apoplastically from the xylem to all leaf tissues [9] (Figure 1). The pattern of Sr distribution in the leaves of *Citrus jambhiri* was similar to that of Ca, suggesting common transport pathways [9]. In contrast, scanning electron microscopy with energy-dispersive X-ray analysis revealed that in the leaves of *Lactuca sativa*, the weight percentage of Sr in the mesophyll was lower than that in the vascular bundle sheath [22]. In the leaves of *Zea mays* seedlings, Sr was found in all tissues, and its preferential accumulation in particular tissues was not observed [100]. No differences between Sr accumulation in the epidermis and mesophyll were observed in *Plantago major* [44]. This suggests that the mechanisms of Sr transport are similar, but their efficiency may differ between cell types and tissues, resulting in interspecific differences in the pattern of Sr accumulation.

Using an energy-dispersive X-ray microanalysis system equipped with a variable-pressure scanning electron microscope, in the leaves of *Morus alba*, Sr was detected in specialized cells called idioblasts, which are distributed in the epidermis. The subcellular compartmentation of Sr completely overlapped Ca localization [115]. There are two types of Ca deposition in idioblasts, i.e., CaCO_3_ and Ca oxalate [115], and the latter plays an important role in the detoxification of various metals, including Sr, by their incorporation into oxalate crystals [8,9,116,117,118]. Strontium oxalate and sulfate crystals were found mainly in the cell vacuoles in phyllodes of the four *Acacia* species. Strontium-containing crystals were most likely precipitated in the epidermis, mesophyll, parenchyma, sclerenchyma, and some other tissues, which may be considered not only an effective Sr detoxification mechanism but also a means of protecting plants from herbivory [119].

The patterns of Sr distribution over root and shoot tissues in plants capable of hyperaccumulating other elements is of particular interest. In the roots of the Zn/Cd hyperaccumulator *Arabidopsis halleri*, at a low Sr concentration (0.1 mM), the Sr content in the central cylinder was higher than that in the cortex, while the opposite pattern was found at 1 mM Sr in the medium. In the leaves, a higher Sr content in the epidermis compared to the mesophyll was found only at 100 mM Sr, whereas no differences were observed at lower Sr concentrations in the medium [82].

Using scanning electron microscopy-energy dispersive X-ray spectroscopy, Sr accumulation was shown for leaf trichomes of *Arabidopsis halleri* exposed to high Sr levels in the medium [82]. Accumulation of Cd, Ni, and Zn in leaf trichomes has been shown for different Brassicaceae species [58], indicating the absence of metal specificity of this phenomenon. Metal accumulation in leaf epidermis may result from their passive translocation along with the transpiration-driven water flow, which terminates in the epidermis [120]. Obviously, this phenomenon may be associated with metal sequestration in cells with low metabolic activity [121].

The high mobility of Sr and its localization in all tissues and organs of plants determines its influence on various physiological processes.

## 5. The Effects of Sr on Enzyme Activities

Alterations in enzyme activities reflect multiple toxic effects of Sr (Table 1 and Table 2). Metals can displace essential elements from metalloenzymes, bind to functional groups in the enzyme reaction center, as well as to groups responsible for stabilizing the protein structure. This results in a change in the enzyme conformation, which is accompanied by a decrease in its activity [5,122,123].

Strontium can induce oxidative stress leading to the accumulation of reactive oxygen species (ROS), the induction of the activities of several antioxidant enzymes (Table 2), and the increase in the production of secondary metabolites [18,126]. In plant cells, ROS are formed in peroxisomes, mitochondria, and chloroplasts [127]. The generation of ROS is a prevalent phenomenon under abiotic and biotic stresses. ROS can attack polyunsaturated fatty acids, which results in lipid peroxidation and destruction of membrane integrity and functioning. Strontium-induced increase in the level of malondialdehyde, a biomarker of oxidative stress, has been demonstrated in *Amaranthus mangostanus* [81], *Avena sativa* [66], *Cucumis sativus* [83], *Lactuca sativa* [22], *Oryza sativa* [57], *Populus russki* [69], *Raphanus sativus* [77], as well as a moss species *Racomitrium japonicum* [124].

The development of oxidative stress is largely attributed to a metal-induced imbalance between the generation of toxic oxygen radicals and their scavenging through the antioxidant defense mechanisms. The latter provide an efficient system for ROS detoxification involving antioxidant enzymes (ascorbate peroxidase, monodehydroascorbate reductase, dehydroascorbate reductase, glutathione reductase, glutathione peroxidase, glutathione S-transferase, superoxide dismutase, peroxidase, catalase) and non-enzymatic antioxidants (ascorbate, anthocyanins, glutathione, phenols, flavonoids). Enzymatic and non-enzymatic mechanisms work synergistically to scavenge ROS and protect plant cells from oxidative damage [127].

Under the influence of Sr, the activity of antioxidant enzymes can either increase [18,45,46,66,77,81,83,97,124,125] or decrease [66,69,77,81,97] (Table 2). For example, after 30 days of growth of *Raphanus sativus* on soil amended with Sr (5–40 mg kg^−1^), catalase activity increased at 5 and 10 mg kg^−1^ Sr, whereas at a higher Sr content, it slightly decreased. After 60 days of treatment, catalase activity sharply decreased compared to the control in a concentration-dependent manner [77]. The activities of catalase, peroxidase, and superoxide dismutase in the leaves of *Lactuca sativa* exposed to Sr changed differently depending on the duration of exposure [22]. In general, enhancement or suppression of antioxidant enzyme activities in the presence of Sr may be induced depending on the metal concentration, duration of treatment, plant tissue or organ, as well as interspecific and intraspecific differences, which determine the efficiency of ROS detoxification mechanisms and, consequently, plant tolerance to metal-induced oxidative stress [5]. A decrease in the activity of antioxidant enzymes at high Sr levels in the medium or during long-term treatments can lead to increased oxidative stress and metabolic disorders in plants.

Since many antioxidant enzymes are metalloenzymes, their activity can decrease as a result of either the displacement of essential elements or metal-induced deficiency of the mineral elements they contain. The displacement of essential elements from metalloproteins under the influence of Sr has not been directly demonstrated, while a Sr-induced decrease in the content of, for example, Fe has been observed in the roots and shoots of various plant species [20]. The increase in the activity of these enzymes observed under the influence of Sr (Table 2) may be due to metal-induced ROS formation.

Metal-induced ROS formation leads to protein degradation [18,46,72], which can ultimately lead to an increase in the content of amino acids, in particular that of proline. When 10-day-old *Amaranthus caudatus* plants were growth in soil amended with Sr (2–10 mM) for 7 days, a concentration-dependent increase in the contents of amino acids, including proline, in the leaves was observed [18]. An increase in proline levels was also shown for *Cucumis sativus* at 10–160 mg kg^−1^ Sr in soil [83] and when cuttings of *Populus alba* and *Populus russkii* were grown at 10 and 100 mM Sr for 3 months [69]. Proline not only protects cells from Sr-induced oxidative damage but also plays an osmoprotective role to regulate macromolecule stabilization and water balance [128,129].

Plant secondary metabolites such as phenolics and flavonoids, including anthocyanins, are also essential bioactive phytochemicals with significant ROS scavenging and metal-chelating properties [130,131]. An increase in anthocyanin content was observed in *Amaranthus caudatus* leaves at 2–10 mM Sr [18] and in the kernels of Sr-treated *Zea mays* plants [132], whereas an increase in total phenolic content in the shoots of *Hypericum perforatum* was shown at 100 µM Sr in the medium [39]. At the same time, the phenolic content in the roots of Sr-treated *Hypericum perforatum* did not change significantly [39]. It was later shown that Sr can affect the phenylpropanoid-flavonoid pathways, enhancing anthocyanin biosynthesis predominantly via the upregulation of the genes encoding the key rate-limiting enzymes such as phenylalanine ammonia-lyase (*PAL*), cinnamate 4-hydroxylase (*C4H*) and dihydroflavonol 4-reductase (*DFR*) [132]. Strontium-induced increase in anthocyanin content [18] and phenolic content [39] may promote metal binding, which in turn decreases the accumulation of ROS and minimizes oxidative stress in metal-treated plants [130]. Interestingly, no significant increase in ROS formation was observed in *Hypericum perforatum* even at 100 µM Sr [39].

## 6. The Effects of Sr on Mineral Nutrition

Since macro- and microelements play an important role in plant cell metabolism, metal-induced disturbance of mineral nutrition leads to the manifestation of multiple toxic effects (Table 1) [4,5,123,133]. Data on the effects of Sr on plant ionome are scarce. Sr may affect the uptake and transport of other ions through multiple mechanisms based on the similarity of metal ion radii, non-specificity of transport pathways and Sr-induced metabolic disorders. Calcium and K carriers may be involved in Sr transport [7], which results in competition between Sr and these elements during their uptake and transport. Therefore, with an increase in Sr content in the growth medium, the contents of Ca and K often decreased, although not in all organs and not at all Sr levels [20,22,25,26,43,44,45,46]. Organ-specific response was shown, for example, for *Zea mays*, in which Ca content in roots decreased at 0.1–10 mM Sr, whereas Ca content in shoots lowered only at 1 and 10 mM Sr [13]. In *Glycine max*, Ca accumulation decreased in the roots at 0.5–4 mM Sr, did not change in the stems or seeds, but increased in the leaves at 4 mM Sr [25]. On the contrary, there was an increase in Ca accumulation in the roots of *Eichhornia crassipes* and *Monochoria hastata* at 0.3–3 mM Sr, whereas Ca content in their leaves fluctuated [72].

The effect of Sr treatment depends on the Sr concentration, sometimes in a species-specific way. For example, in *Brassica rapa*, the Ca content in leaves did not change at 0.1–1 mM Sr [28], but decreased at 2–10 mM Sr [26]. Similarly, in *Vicia faba*, the Ca content in leaves decreased only at higher Sr concentrations (5.86 and 11.36 mM) [45]. However, in *Alstonia scholaris*, the Ca content in leaves decreased only at lower Sr concentrations (0.3 and 1.3 mM), but did not change at a higher one (3 mM) [46]. It is difficult to explain this discrepancy, as it may be due to either species-specific plant response or different experimental conditions, such as, e.g., growth medium type (soil/nutrient solution), nutrient composition of growth medium, Ca and Sr concentrations, Sr salt used, duration of exposure, plant developmental stage.

Although the Ca:Sr ratio is an important parameter, in the majority of works studying the effects of Sr on Ca uptake and accumulation, only one concentration of Ca was tested, and the equimolar Ca and Sr concentrations were almost never used. So, this parameter is rarely evaluated. For example, in the two studies conducted under identical growth conditions, at 0.01–100 mM Sr in the nutrient solution, the Ca:Sr ratio was of similar magnitude in the leaves of *Plantago major* [44], whereas in the leaves of *Arabidopsis halleri*, it fluctuated at 0.01–1 mM and significantly increased at 10 and 100 mM Sr [82]. Interestingly, at equimolar Sr and Ca concentrations in the solution (1 mM), the Ca:Sr ratio was around 290 in *Plantago major* [44] and around 2.3 in *Arabidopsis halleri* [82]. Species-specific response may be related to the plant type, i.e., whether it is a calcicole species preferentially growing on calcareous soils, a calcifuge species avoiding calcareous soils, or a soil-indifferent plant species, as the patterns of Ca uptake, transport and accumulation may differ in them [134]. However, these plant types have never been compared in relation to the effect of Sr. Therefore, more detailed studies are needed to elucidate the effects of Sr on plant mineral nutrition. As the differences in the observed trends may also be related to different experimental conditions, which hinder the comparison of the results obtained in different works, it is crucial to perform such comparative studies under identical growth conditions at a wide range of Sr and Ca concentrations in the medium, testing the initial Ca level in plant as well.

Under the action of Sr, the contents of particular nutrients can remain constant or change to varying degrees depending not only on the Sr concentration [20,21] but also on the duration of treatment [22]. For example, Mg, sulphur (S) and phosphorus (P) accumulation in the leaves of *Lactuca sativa* did not change significantly after 21 days of incubation in the presence of Sr, while the contents of Ca and Fe decreased, and that of K increased. However, after 7 and 14 days of treatment, there was no significant effect of Sr on Fe and K contents in leaves yet [22].

Obviously, as the analogue of Ca, Sr can inhibit Ca-dependent cellular processes via competition with and replacement of Ca [7]. Disruption of mineral nutrition at supra-optimal Sr concentrations in the environment can significantly affect the physiological state of plants, ultimately leading to a disruption of photosynthesis and growth.

## 7. The Effects of Sr on Photosynthesis

Being accumulated in leaves, Sr enters the chloroplasts and can induce significant structural and functional alterations [135], leading to the disruption of various photosynthetic processes, such as energy absorption, energy transfer, and photosynthetic carbon assimilation [74]. Disruption of photosynthetic processes negatively impacts plant growth, yield, and productivity (Table 1).

A decrease in the contents of chlorophyll and carotenoids is a typical consequence of the toxic effects of various metals, which is often observed during plant cultivation both hydroponically and in soil [4,5,123,133]. A Sr-induced decrease in chlorophyll content was observed in hydroponically grown *Alstonia scholaris* [46], *Amaranthus caudatus* [18], *Arabidopsis halleri* [82], *Brassica napus* [74], *Glycine max* [24], *Lactuca sativa* [22], *Monochoria hastata* [72], *Morus alba* [115], *Vicia faba* [45], as well as in *Amaranthus mangostanus* at 1 and 5 mmol kg^−1^ Sr in soil [81], *Avena sativa* (Bayou-3 genotype) at 500 mg kg^−1^ Sr in soil [66], *Cucumis sativus* at 40–160 mg kg^−1^ Sr in soil [83], and *Raphanus sativus* at 5–40 mg kg^−1^ Sr in soil after 60 days of treatment [77]. The content of carotenoids in leaves decreased after 21 days of growing *Lactuca sativa* at 3 mM Sr [22], and after 7 days of incubation of *Amaranthus caudatus* in the presence of 2–10 mM Sr [18] (Table 3, Figure 2).

The effect of Sr on the contents of photosynthetic pigments may vary depending on its concentration in the medium. At low Sr level in the medium (0.01 mM), the chlorophyll content did not change in the excluder *Glycine max* grown in soil [43] or in the hydroponically grown Zn/Cd hyperaccumulator *Arabidopsis halleri* [82]. An increase in the chlorophyll content was observed in the leaves of *Brassica rapa* at 0.1–1 mM Sr [26,28], as well as at 10 mM Sr in *Beta vulgaris* [88] and *Brassica napus* [74], which can enhance light absorption by plants. The mechanism behind this phenomenon is not yet fully understood, but may be partly related to an increase in the contents of Mg, K, and P, which was observed, for example, in *Lactuca sativa* after a 21-day exposure to 1–5 mM Sr [22]. However, in the leaves of *Zea mays* and *Amaranthus caudatus*, at the same concentration (10 mM Sr) in the medium, there was a decrease in chlorophyll content [13,18], whereas in soil-grown *Trifolium pratense*, the contents of chlorophyll *a* and chlorophyll *b* did not change at 5–40 mM Sr [78], indicating that its effect is species-specific. In *Brassica napus* [74], *Glycine max* [24], *Vicia faba* [45], and *Zea mays* [13], chlorophyll *a* content was more sensitive to Sr than chlorophyll *b* content. Other species showed a different trend [22,137], which, as for other metals [5], may be determined by the differences in metal concentration, duration of treatment, plant age, as well as interspecific and intraspecific characteristics. For example, at 4 mM Sr, the contents of chlorophylls *a* and *b* decreased by approximately 50% in young leaves of *Morus alba*, whereas the chlorophyll content in mature leaves remained virtually unchanged compared to untreated plants [115] (Table 3). In some cases, the decrease in chlorophyll content in leaves was accompanied by pronounced signs of chlorosis and necrosis [46].

The decrease in chlorophyll content can be determined by various mechanisms, including Sr-induced deficiency of Fe [20,39] and Mg [13,20,39,45,46], the production of H_2_O_2_ [66,124], which resulted in chloroplast membrane peroxidation, as well as the inhibition of enzymes involved in chlorophyll biosynthesis, which has been shown for other metals [5,123] (Figure 2).

Stable Sr at elevated concentrations can affect the light and dark reactions of photosynthesis. Some researchers have reported Sr-induced changes in the stomatal (net photosynthetic rate (*P*_n_), intracellular CO_2_ concentration (*C*_i_), stomatal conductance (*g*_s_), and transpiration rate (*E*)) and non-stomatal (photosystem II activity (*F*_v_/*F*_m_), photochemical quenching (qP), electron transport rate (*ETR*) or maximum light-driven electron transport rate (*J*_max_)) attributes of photosynthesis [28,45,97,136,137] (Table 3).

In *Arabidopsis halleri* (at 0.1–100 mM Sr) [82], *Plantago major* (at 0.01, 1, 100 mM Sr) [44] and *Salix babylonica* (at 8 mM Sr) [136] a significant reduction in maximum photochemical efficiency of photosystem II (PSII, *F*v/*F*m) was observed, indicating inhibition of light absorption and energy accumulation in the antenna complex. The down-regulation of PSII caused by Sr was observed for the leaves of *Brassica napus* at 40 mM Sr [74] and *Zea mays* at 0.2 mM Sr [138]. Under moderate and high Sr-induced stress, photo-inhibition and photo-damage in PSI and PSII was shown for *Racomitrium japonicum* [124]. In *Amaranthus tricolor* treated with 6 mM Sr, the acceptor side of PSII was more sensitive to Sr stress than the donor side. Strontium accumulation led to disturbed electron transport in PSII in the leaves of *Amaranthus tricolor* [27]. Calcium ions are essential for oxygen evolving complex to maintain efficient performance. Strontium ions can substitute Ca ions in the PSII oxygen evolving complex, disrupting its activity [139,140]. For example, Gates et al. [141] revealed lower oxygen evolution activity of PSII(Sr) compared to PSII(Ca). Disturbance in leaf photosynthetic oxygen evolution rate and decrease in the activity of the key photosynthetic enzyme Rubisco and phosphoenolpyruvate carboxylase, as observed in Sr-treated *Brassica napus*, increased with the duration of treatment and Sr concentration in the medium [74]. Reduced photosynthetic intensity usually leads to an excess of light energy, which produces toxic ROS in plant cells. The ROS produced within PSI cannot be immediately scavenged by the antioxidant system, leading to oxidative damage to PSI and disrupted linear and cyclic electron transport. To diminish oxidative stress, on the one hand, plants dissipate excess light energy as heat by increasing non-photochemical quenching (NPQ), which is a photoprotective mechanism aimed at preventing the formation of ROS, and on the other hand, they remove excess ROS by increasing the activities of antioxidant enzymes [45,124] (Table 2 and Table 3, Figure 2).

Plant treatment with Sr inhibited stomata opening [13] and led to decreased stomatal density, stomatal aperture [45], and stomatal conductance [11,45], resulting in a limited supply of carbon dioxide necessary for carbon fixation. Metal-induced decrease in leaf area [18,26,40,45,69,83] and number of leaves [40,43], the main photosynthetic organs, also contributes to the decrease in the efficiency of photosynthesis under the influence of Sr (Figure 2).

However, at low Sr concentrations, the light reaction rate was accelerated, the energy utilization efficiency of the photosystems (PSI and PSII) was improved and ultimately the photosynthetic performance of *Brassica rapa* leaves was enhanced, which was accompanied by a significant increase in starch content and leaf thickness [28]. Low Sr concentrations (2.84 mM) also promoted the photochemical efficiency and electron transfer of PSII in the leaves of *Vicia faba* [45]. The increased electron transfer efficiency in the donor side of PSII at low Sr concentration in the medium was confirmed by Chao et al. [28]. The data obtained on *Brassica rapa* are consistent with an increase in chlorophyll content, leaf area, leaf water content, and growth stimulation at low Sr levels [26,28].

## 8. The Effects of Sr on Plant Growth and Morphogenesis

Impaired growth and morphogenesis is one of the most important visible symptoms of metal-induced stress, so it is often used to assess metal toxicity [5,6,123,133]. Growth processes are a very useful and interesting model that allows us to examine the relationship between the patterns of metal distribution and the mechanisms of their toxic effects. In an early study, Brooks [142] estimated that the toxicity level of Sr for plants is 30 ppm. Later, a series of studies was conducted to comprehensively evaluate its toxic effect on plant growth (Table 1).

At elevated concentrations, Sr [102,125,143,144], as well as other metals [5,102,123,133], usually inhibit the germination of seeds and caryopses (Figure 3). Radicle emergence is generally more tolerant to the effects of metals than subsequent germination involving radicle growth and shoot emergence, as metals can freely penetrate embryonic tissues once the integrity of the protective coverings is breached. However, despite the entry of Sr into the embryonic tissues and endosperm cells of *Zea mays* caryopses, no significant inhibition of radicle emergence and further germination was observed at 10 mM Sr. The latter may be attributed to both the accumulation of Sr predominantly in the apoplast of embryo cells and the relatively low toxicity of this metal. The toxic effect of metals on the germination of caryopses decreased in the series: Cd > Ni ≈ Pb > Sr [102]. Germination of *Arabidopsis thaliana* seeds on agar was inhibited at Sr concentrations exceeding 50 mM [143]. Germination of dimorphic seeds of the salt-tolerant halophyte *Salicornia europaea*, which differ in size, position, and germination behavior, was found to be sensitive to Sr to varying degrees. Germination of large median seeds was not significantly inhibited at Sr concentrations up to 300 mM Sr and reached 84% at 400 mM Sr, whereas germination of small lateral seeds was inhibited already at 200 mM Sr, and at 400 mM Sr, the seeds did not germinate [144]. Interestingly, Sr accumulation in seeds may depend on their size. Larger grains of *Aegilops speltoides* were shown to contain more Sr (mg kg^−1^ dry weight) than smaller ones, and a negative correlation was found between the metal content and 1000-grain weight. Sr accumulation in grains also was shown to depend on climatic conditions [67].

Despite the wide range of environmental Sr concentrations and the relatively low toxicity of this metal (Table 1 and Table 4), selecting an appropriate concentration of the Sr salt is a crucial step when planning experiments with either seedlings or mature plants. Natural background levels of stable Sr in soil typically range from around 20 up to around 260 mg kg^−1^ in non-contaminated soils and up to 1000–3000 mg kg^−1^ in heavy loamy soils [19]. Thus, environmentally relevant Sr exposure levels for the non-hypertolerant plant species should fall within this limit, preferably closer to the range of 20–260 mg kg^−1^. Plant cuttings cannot be considered an optimal model for physiological experiments, as metal uptake may occur not only through developing adventitious roots but also through the cut surface. Since the manifestation of the metal toxic effects depends on a multitude of factors, such as metal concentration, growth medium, growth conditions and the specific plant characteristics (cultivar, species, age, stage of development, etc.), a detailed assessment of toxicity must be conducted on a case-by-case basis when determining working concentrations. To assess the toxic effect of Sr, as well as other metals, various plant growth parameters are used, including changes in root and shoot length and biomass, leaf area, leaf number, etc. [11,24,26,27,40,44,71,82,83,84,100,104,115,143,145,146] (Table 4, Figure 3). In addition, visible toxicity symptoms such as wilting, leaf chlorosis and necrosis, and browning of root tips [5] are used, as well as the changes in the contents of photosynthetic pigments and the concentrations of malondialdehyde (MDA) or thiobarbituric acid reactive substances (TBARS) as biomarkers of oxidative stress, and other biochemical parameters (Table 1).

Since metals enter plants primarily through the root system, the primary response to their exposure is observed in the roots. Therefore, root growth is widely used as a test system for assessing the toxic effects of metals, especially in hydroponics [6,147]. For this purpose, the metal concentration that causes 50% inhibition of root growth (EC_50_) is often determined [148,149,150]. To assess the toxic effect of a metal on root growth in mature plants, a sequential exposure test can be applied. A series of linearly increasing metal concentrations is tested against a background of nutrient solution until root growth ceases completely. At the start of the experiment and immediately before each increase in metal concentration, the root systems are stained black by immersion in a charcoal suspension. If the roots continue growing, unstained root apices become visible. The test is finished at the metal concentration at which unstained root apices are no longer visible, which indicates root growth arrest. This concentration value (EC_100_) may serve as a criterion for assessing plant tolerance. Obviously, the lowest effective concentration, the EC_50_ and EC_100_ values, as well as metal concentration imposing a lethal effect (LC_100_), and the range between them can vary significantly depending on the metal, plant species, and population. This is particularly evident when comparing excluder and hyperaccumulator species, which differ drastically in their metal tolerance and accumulation capacities [151]. Determining the EC_50_ and the ratio between the metal concentration used in the experiments and the EC_100_ is crucial, although it is rarely done in physiological studies as it is highly labour-consuming.

For *Zea mays*, the toxicity of metals, assessed by root length increment over 48–72 h of treatment with metals at the concentration of 0.01 g L^−1^, decreased in the following order: Cu ≈ Tl > Ag > Cd > Hg > Co ≈ Ni > Zn > Pb > Sr. A similar toxicity series was obtained when metal concentrations causing 50% root growth inhibition (EC_50_) were used as the evaluation criterion. At the same molar concentration of metals in the solution, e.g., 0.1 mM, their toxicity decreased in the following order: Tl^3+^ > Cu^2+^ > Ag^+^ > Hg^2+^ ≈ Cd^2+^ > Ni^2+^ > Zn^2+^ ≈ Pb^2+^ ≈ Co^2+^ > Sr^2+^. Since different metals differ in atomic weight, it is more appropriate to compare molar rather than weight concentrations, although this is not always done. However, regardless of the analytical method, among the studied metals, Sr was the least toxic [102,113,147]. Kopittke et al. [152] conducted a meta-analysis of the data published in the literature and obtained the following median toxic concentrations of trace metals in nutrient solutions, which increased in the row Pb (0.30 μM) ≈ Hg (0.47 μM) < Cu (2.0 μM) < Cd (5.0 μM) ≈ arsenate [As(V)] (9.0 μM) < Co (17 μM) ≈ Ni (19 μM) ≈ Zn (25 μM) < Mn (46 μM). Thus, metal toxicity decreased in the order Pb ≈ Hg > Cu > Cd ≈ As(V) > Co ≈ Ni ≈ Zn > Mn [152]. Strontium was not included in the list of elements that were analyzed for toxicity by these authors. However, the analysis of the literature data presented in Table 1 and Table 4 shows that toxic concentrations of stable Sr are usually in the millimolar range, which is significantly higher than those for many other metals. However, metal toxicity ranges can vary widely depending on plant growth conditions, metal concentrations, duration of treatment, plant age, as well as plant species or genotypes [5,6,133,152].

At low concentrations, Sr may stimulate plant growth, whereas at high concentrations, growth inhibition is observed, which reflects its dual effects. For example, at 1.5 mM Sr, stimulation of root and shoot growth of *Glycine max* was observed, whereas at 3 mM Sr, there was a significant decrease in fresh and dry biomass of both organs [24]. At the same time, Dresler et al. [25] observed an increase in root biomass accumulation in *Glycine max* treated with 4 mM Sr compared to the control, when Sr content in roots reached 5 mg g^−1^ dry weight. This once again confirms the difficulty of comparing the results obtained by different authors due to the above-mentioned reasons. Growth stimulation at low Sr concentrations (0.1–2 mM) was observed in different species and usually did not exceed 20% of the control [22,23,26,27]. In *Brassica rapa*, growth-stimulatory effect was manifested as an increase in specific leaf weight in Sr-treated plants compared to control plants [28]. Meanwhile, in *Amaranthus tricolor*, at 0.2 mM Sr, shoot dry weight increased by approximately 36%, and root dry weight increased by 60% [27], indicating interspecific differences in plant growth response to Sr.

Plant age also influences the concentration-dependent Sr-induced growth response. In *Zea mays* seedlings treated with 10 mM Sr for 24 and 48 h, the radicle growth was significantly inhibited, but the root growth of 7-day-old plants treated with 10 mM Sr for 7 days was not reduced, whereas at 0.1 mM Sr, there was no significant effect on the growth of 4 mm-long seedling radicles but the growth of older roots was stimulated [13].

Plant growth conditions also significantly influence the toxic effects of metals. For example, the growth-inhibitory effect of Sr on *Arabidopsis thaliana* grown on agar medium was first detected at 20 mM Sr and increased with the increase in Sr concentration up to 40 mM [143], whereas for the plants of other species grown hydroponically, the toxic effect of Sr was observed at much lower concentrations in the medium (Table 4). These differences may be determined not only by the different tolerance to Sr among the species but also by the metal binding to agar, resulting in the effective concentration of Sr being significantly lower than stated. Similar patterns may also be observed when growing plants in roll culture, as well as in Petri dishes on filter paper moistened with solutions of metal salts. Due to the significant metal sorption by filter paper, as well as only partial root contact with the nutrient solution, metal uptake by plants can be significantly reduced. Furthermore, this method of growing seedlings leads to a production of a large number of root hairs, making it difficult to separate the root system from the filter paper when preparing plant material for metal quantification. Metal accumulation in plants grown hydroponically and in soil can also vary significantly, due to soil heterogeneity, uneven metal distribution across the soil profile, and a different ratio of macro- and microelements compared to a solution. Consequently, even when identical amounts of a metal are added to the soil and the nutrient solution, the quantity of metal available to plants can differ significantly, depending on the composition of the nutrient solution and the physicochemical properties of the soil. For example, following a single soil amendment with Sr, even at a relatively high concentration, the amount of plant-available Sr in the soil may be considerably lower than that in the nutrient solution due to its binding with the components of the soil adsorption complex. Unfortunately, laboratory experiments involving soil-grown plants often fail to assess the amount of plant-available metal, reporting instead only the total Sr content, which may even fall within the range of natural Sr levels found in various soil types. Therefore, when planning an experiment, it is important to determine the plant-available metal content and conduct preliminary testing of the metal toxic effects based on a comprehensive set of parameters, utilizing a range of metal salt concentrations for each specific case.

The manifestation of growth-inhibiting effects depends not only on the chosen metal concentration in the medium but also on the metal content and distribution within the plant, including one in the growing regions of the root and shoot [103]. Consequently, a comprehensive analysis of the metal toxic effects is only possible when the data on metal content and distribution across plant tissues are available.

Therefore, the use of varying growth conditions (growth media, metal concentrations, duration of treatment, etc.), different metal salts, and plants at different developmental stages, taken altogether with inter- and intraspecific differences, can make it extremely difficult to compare data obtained in different works. A valid comparison of results is only possible when plants of the same age are grown under identical conditions. Furthermore, when comparing different metals, identical molar concentrations rather than mass-based concentrations should be used.

Metal treatment can significantly alter root system architecture, which depends on the degree of development of roots of different types. The development of a branched root system with a relatively large number of younger roots and roots of higher branching orders can affect plant mineral nutrition. Changes in the orientation, branching, and secondary growth of the main root types lead to a significant diversity in root system architecture in different plant species [153,154]. Root branching occurs due to the formation of lateral roots above the elongation zone. Lateral root formation occurs through cell division in the pericycle. The time it takes for lateral roots to form, from the first pericycle cell division to the emergence of lateral roots from the parent root, is generally tolerant to the effects of various metals [147]. Despite the Sr entry into pericycle cells, lateral roots in *Zea mays* seedlings grown in the presence of Sr emerged approximately 50 h after incubation, similarly to control roots [100,103]. However, in Sr-treated *Vicia faba*, not only taproot length but also lateral root number decreased, which affected root system architecture [45].

Root growth is determined by two processes: cell division in the meristem and cell elongation in the elongation zone [6]. An integrative analysis examines metal growth-inhibitory effects depending on the duration of exposure, and also evaluates the meristem length, the number of cells in the meristem, the mitotic index (MI) (the proportion of meristematic cells that proliferate), the cell cycle duration, the relative rates of cell production and elongation, the length of fully elongated cells, as well as cell viability and other parameters [155,156,157].

Upon entering the meristem, Sr inhibits cell division, which is accompanied by a decrease in the MI. The latter may be due to a prolongation of the cell cycle. For example, the duration of the cell cycle increased twofold in the roots of *Zea mays* seedlings on the second day of treatment with Sr. Inhibition of cell division was accompanied by a decrease in the number of cells in the meristem and meristem length [84]. A decrease in the MI under the action of Sr in a time- and concentration-dependent manner was observed in the root tips of *Allium cepa* [146] and *Vicia faba* [158]. In contrast to the meristem, Sr-induced decrease in the MI was not detected in the root cap [104] (Figure 3).

The negative effect of Sr on the MI is likely to result from Sr-induced mitotic abnormalities in dividing cells of the root meristem. Allium test is the most common test used to determine the cyto- and genotoxicity of various metals because *Allium cepa* cells have a small number of relatively large chromosomes which can be easily observed under the microscope under laboratory conditions [5]. Strontium-induced C-metaphases, lagging chromosomes, multipolar anaphases, chromosomal stickiness and chromosomal aberrations such as bridges and breaks were observed in the root tips of *Allium cepa* [146]. In root tip cells of *Vicia faba*, the frequency of appearance of chromosomal aberrations and micronuclei increased in the presence of Sr, indicating the structural damage of DNA [158]. Chromosome adherence, chromosome breaking, chromatin bridges, chromosome dispersion, chromosome shrinking, and ring chromosomes were observed in the root tips of *Lens culinaris* exposed to high levels of Sr [159]. These abnormalities, which develop due to Sr accumulation in the root meristem, indicate its clastogenic potential [146] (Figure 3).

A distinctive feature of the effect of Sr on cell elongation is the increase in the length of fully elongated cells [84]. The presence of polysaccharides in plant cell wall offers binding sites for divalent and trivalent metals. In the cell walls, Sr is likely to compete with Ca [11] for binding to polygalacturonic acids, resulting in altered cell wall plasticity. As a result, shapeless cells are often observed in the apical part of *Zea mays* roots, cell rows in the meristem are disrupted, and uneven Sr-induced cell elongation occurs [84,104] (Figure 3). The mitigating effect of Ca supply is clearly evident from a decrease in the influence of Sr on cell division and elongation, which may be due to competition for common binding sites and a decrease in Sr accumulation in the apical part of the root, resulting in a markedly reduced growth-inhibitory effect [84,104]. Calcium supply also alleviated the Sr-induced growth inhibition in *Oryza sativa* cultivars [107].

Plants have to tightly control their growth and development for their survival under severe environmental conditions. However, the molecular mechanisms underlying plant response to Sr-induced stress remain poorly studied. Significant metabolic changes were evidenced by the results of transcriptomic analysis, which showed that 559 genes were up-regulated and 283 genes were down-regulated in *Arabidopsis thaliana* in response to Sr-induced stress. For example, qRT-PCR analysis revealed the up-regulation of the expression of *GGCT2;1* (Gamma-Glutamyl Cyclotransferase 2;1), encoding the enzyme involved in metal detoxification, since it catalyzes the formation of 5-oxoproline from gamma-glutamyl dipeptides and plays a significant role in glutathione homeostasis, and that of *IRT1* (Iron-Regulated Transporter 1), encoding a non-selective metal transporter required for metal uptake from the soil. The genes related to jasmonic acid biosynthesis and signaling were also significantly up-regulated in Sr-treated seedlings, which might be associated with plant response to Sr-induced stress and play an important role in maintaining proper growth and development in the presence of Sr [160] (Figure 3).

Plant treatment with Sr can also lead to disruption of biogenesis of miRNAs which negatively regulate gene expression at the post-transcriptional level through the cleavage of mRNA targets or the translational repression of target genes, including those that control morphogenesis. For example, Sr treatment might induce flowering by the down-regulation of miR156, which in turn leads to increased expression of *SPL4*, which encodes a member of the SQUAMOSA promoter-binding protein-like (SPL) family of transcription factors involved in accelerating the transition from the juvenile vegetative phase to the adult vegetative phase and transition to reproductive development. In addition, it has been shown that Hyponastic Leaves 1 (HYL1) protein, one of the core components of the microprocessor complex involved in miRNA biogenesis, is essential for plant growth and development under Sr-induced stress. Indeed, *hyl1-2* mutant plants were shown to be more sensitive to Sr than wild-type plants, which can be explained by a general reduction in miRNA biogenesis or a reduced accumulation of specific miRNAs which are essential for Sr tolerance. Thus, Sr-induced inhibitory effects may be at least partly associated with altered miRNA biogenesis caused by the reduction in the content of HYL1 protein [143] (Figure 3).

Thus, the growth-inhibitory effect of Sr is determined both by a general metabolic disturbance and by its direct effect on growth processes. Despite varying degree of the effect of Sr on cell division and elongation, it eventually leads to growth inhibition, the strength of which can be used to assess metal toxicity (Figure 3).

## 9. Conclusions and Future Prospects

Strontium is a non-essential element that is chemically similar to Ca and is used as a tracer for Ca transport in plants. At low Sr levels in the environment, it can stimulate plant growth and development, whereas at high levels, it exerts multiple toxic effects on various physiological processes. Strontium affects enzyme activity, mineral nutrition, and photosynthesis, leading to the disruption of plant growth and morphogenesis. Despite some advances in understanding the mechanisms of Sr uptake and transport, as well as the fundamental patterns of its distribution within plant tissues and organs, the molecular mechanisms of its effect on plants still require further studies. Its impact on plant water balance, respiration, and hormonal balance remains largely unexplored. Little information can be found on the toxic effects of combined exposure to Sr and other metals, making it difficult to predict its impact on plants under polymetallic stress. The mechanisms of the growth-promoting effect of Sr at its low concentrations in the medium, as well as the interspecific and intraspecific characteristics of its dual action at various stages of plant development, remain largely unrevealed. The intracellular distribution of Sr and its detoxification pathways have not been thoroughly studied. Given the similarities between the transport pathways of stable Sr and radioactive Sr, the relevance and practical significance of further studies of these aspects are undeniable. Understanding the mechanisms of transport, detoxification, growth-stimulatory and growth-inhibiting effects of Sr is of great practical importance for agriculture, as well as for the selection of Sr-tolerant and Sr-accumulating plant species for the development of modern approaches to phytoremediation and biofortification.

## Figures and Tables

**Figure 1:**
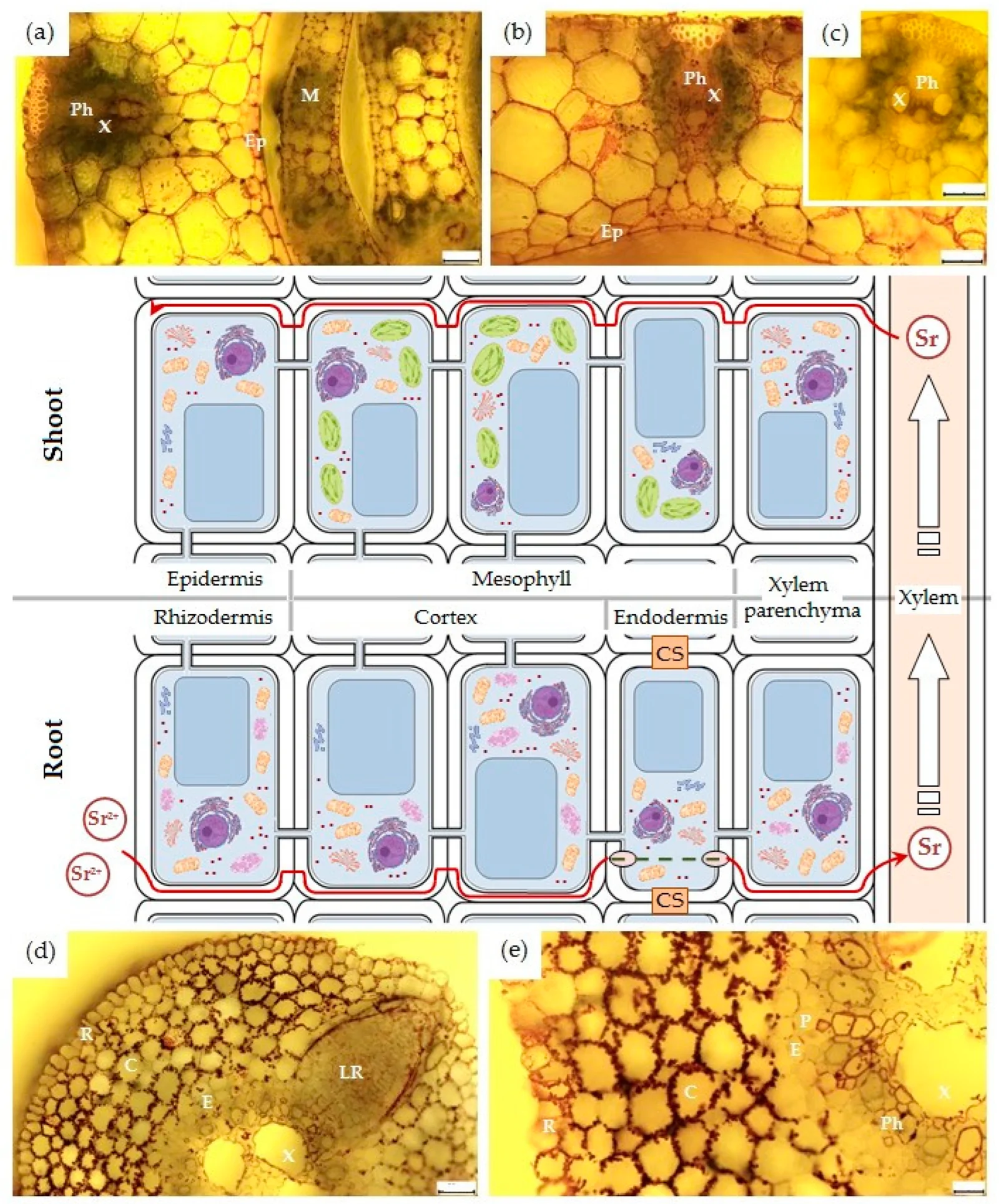
Strontium transport in plant tissues. Strontium distribution in the shoots (**a**,**b**) and roots (**d**,**e**) of *Zea mays* seedlings treated with 3 mM Sr for 6 days. The pictures show histochemical staining with sodium rhodizonate, which produces red–brown complexes with Sr ions. Leaf of untreated plant (control) is shown in section (**c**). (**a**–**c**) leaf cross-sections; (**d**,**e**) mature root cross-sections. Bars, 50 µm (**a**–**d**), 20 µm (**e**). Simplified scheme shows the transport of Sr. The red line indicates Sr transport through the apoplast. The green dashed line indicates its transport through the symplast to bypass the waterproof Casparian strips (CS). White arrows indicate long-distance root-to-shoot Sr transport via the xylem. Strontium entry into the symplast cannot be excluded in tissues other than the root endodermis. C, cortex; E, endodermis; Ep, epidermis; LR, lateral root; M, mesophyll; P, pericycle; Ph, phloem; R, rhizodermis; X, xylem.

**Figure 2:**
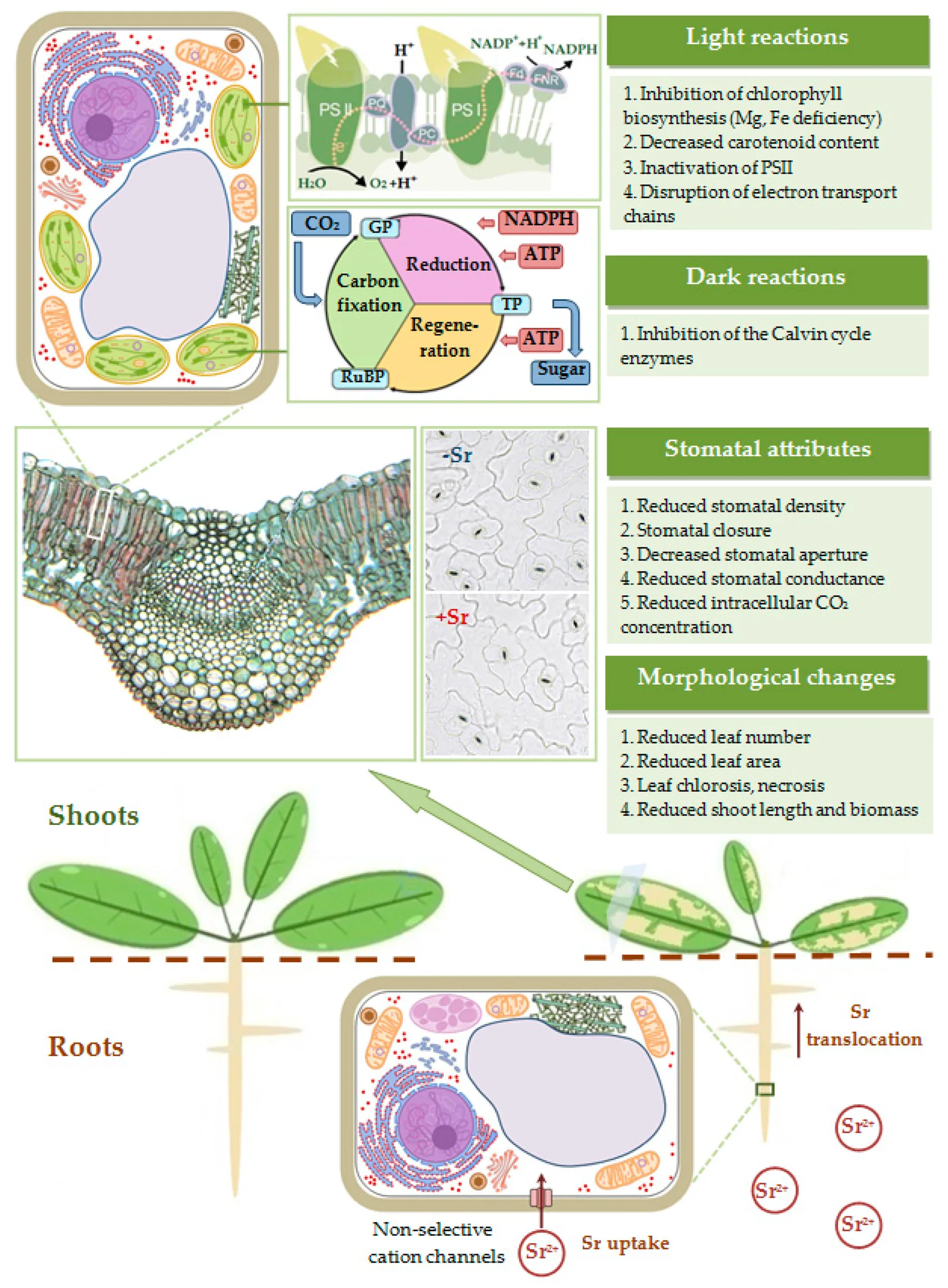
Toxic effects of Sr on photosynthesis. Fd—ferredoxin; FNR—ferredoxin-NADP(H) oxidoreductase; GP—glycerate-3-phosphate; PC—plastocyanin; PSI—photosystem I; PSII—photosystem II; PQ—plastoquinone; RuBP—ribulose bisphosphate; TP—triose phosphate.

**Figure 3:**
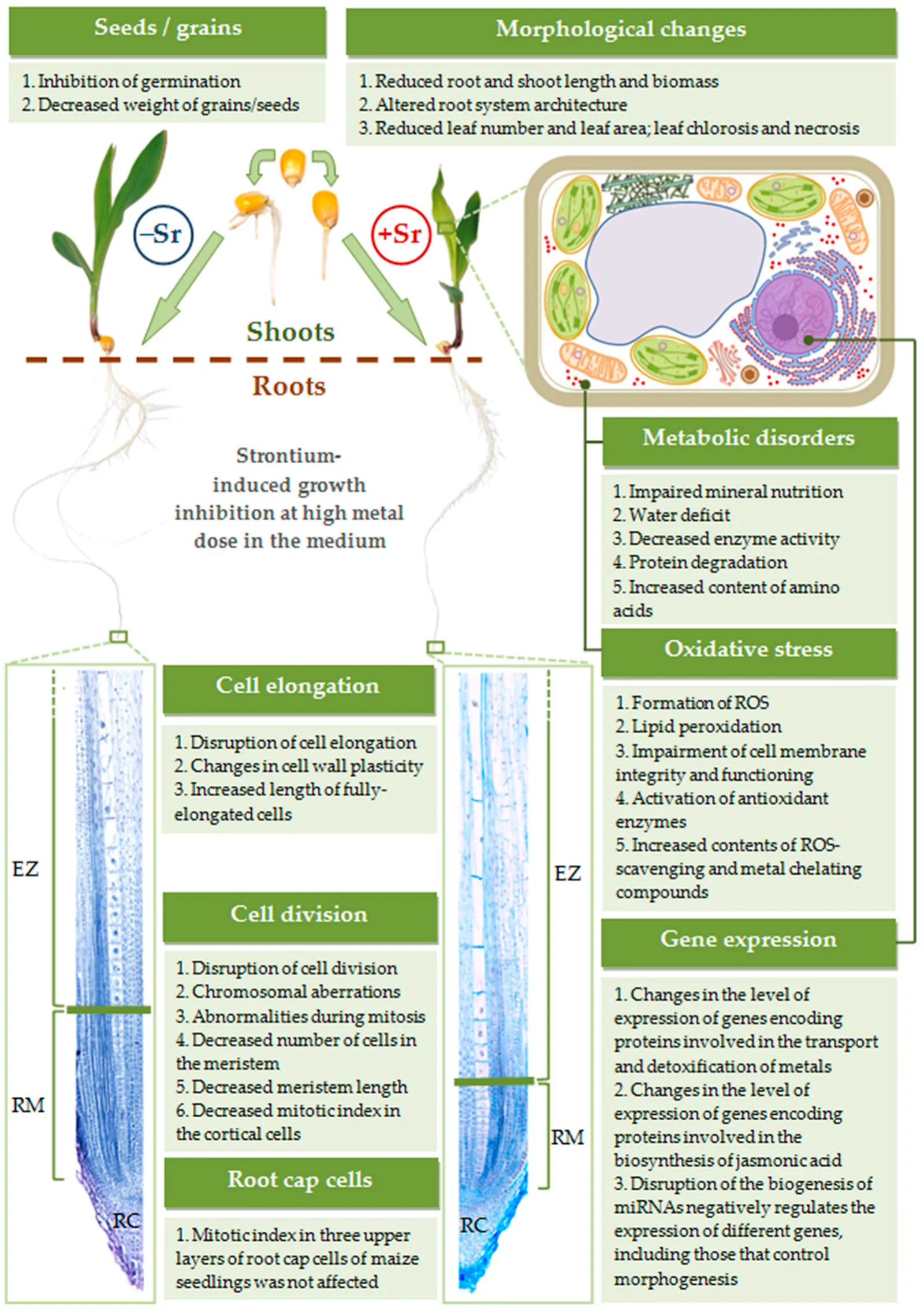
Toxic effects of Sr on plant growth and morphogenesis. EZ—elongation zone; RC—root cap; RM—root meristem; ROS—reactive oxygen species.

**Table 1:** Strontium accumulation in plants and Sr-induced physiological response.

Plant Species	Sr Content/ Concentration	Duration of Exposure	Plant Material	Sr Content in Plants	Physiological Response	Refs.
*Alstonia* *scholaris*	0.3–3 mM in solution	21 days	plants	~1400–8600 mg kg^−1^ dw	Changes in the contents of mineral elements; decrease in chlorophyll and soluble protein contents; induction of activities of antioxidant enzymes; leaf chlorosis and necrosis	[46]
*Amaranthus mangostanus*	1, 5 mmol kg^−1^ in soil	until maturation	roots	201–257 µg g^−1^ dw	Reduction in root and shoot biomass; changes in chlorophyll content and antioxidant enzyme activities	[81]
			shoots	218–283 µg g^−1^ dw		
*Apium* *graveolens*	0.5–10 mM in solution	21 days	roots	~6–37 mg g^−1^ dw	Changes in the contents of mineral elements	[21]
			leaves	~2.5–26 mg g^−1^ dw		
*Arabidopsis halleri*	0.01–100 mM in solution	7 weeks	leaves	46–5166 mg kg^−1^ dw	Decrease in root, leaf dry weight and chlorophyll fluorescence at highest concentration(s)	[82]
*Avena sativa* Bayou-3 genotype	25, 100, 500 mg kg^−1^ in soil	30 days	roots	~60–500 mg kg^−1^ dw	Reduction in root and shoot biomass; decrease in chlorophyll content ^(2)^; increase in malondialdehyde content in leaves ^(2)^ and roots; induction of activities of antioxidant enzymes	[66]
			stems	~70–650 mg kg^−1^ dw		
			leaves	~100–870 mg kg^−1^ dw		
*Avena sativa* Neimengkeyimai-1 genotype	25, 100, 500 mg kg^−1^ in soil	30 days	roots	~80–1600 mg kg^−1^ dw	Increase in malondialdehyde content in leaves ^(2)^ and roots; induction of activities of antioxidant enzymes	[66]
			stems	~70–1100 mg kg^−1^ dw		
			leaves	~90–1700 mg kg^−1^ dw		
*Brassica napus*	10–40 mM in solution	14, 21 days	roots	16–28 g kg^−1^ dw	Decrease in chlorophyll content and photosynthetic enzyme activity	[74]
			stems	2–10 g kg^−1^ dw		
			leaves	18–57 g kg^−1^ dw		
*Brassica rapa*	0.1–1 mmol L^−1^ in solution	28 days	leaves	~1–7 mg g^−1^ dw	Increase in chlorophyll ^(4)^, protein ^(4)^ and starch content in leaves; promoting effect on photosynthetic performance	[28]
	0.2–10 mmol L^−1^ in solution	30 days	leaves	~3–30 mg g^−1^ dw	Increase in protein content in leaves; increase in chlorophyll content at lower Sr doses; changes in water content in leaves; changes in fresh weight	[26]
*Cucumis* *sativus*	20–160 mg kg^−1^ in soil	60 days	roots	342–621 µg g^−1^ dw	Reduction in root and shoot biomass; inhibition of plant growth; decrease in chlorophyll content ^(1)^; oxidative stress ^(1)^; increase in proline content; induction of activities of antioxidant enzymes	[83]
			shoots	226–1197 µg g^−1^ dw		
*Eichhornia crassipes*	0.3–3 mM in solution	30 days	roots	~10–40 g kg^−1^ dw	Changes in the contents of mineral elements; decrease in soluble protein content; induction of activities of antioxidant enzymes; growth inhibition at the highest concentration	[72]
			petioles	~100–130 g kg^−1^ dw		
			leaves	~30–80 g kg^−1^ dw		
*Glycine* *max*	0.5–3 mM in solution	14 days	roots	18–180 mg kg^−1^ dw	Decrease in chlorophyll and carotenoid contents	[24]
			shoots	175–1246 mg kg^−1^ dw		
*Hypericum perforatum*	0.1 mM in solution	7 days	roots	~730 µg g^−1^ dw	Increase in the contents of amino acids in roots and phenolic compounds in shoots; decrease in the contents of most Krebs cycle acids, root ascorbic acid and root and shoot thiols	[39]
			shoots	~400 µg g^−1^ dw		
*Monochoria hastata*	0.3–3 mM in solution	30 days	roots	~10–20 g kg^−1^ dw	Changes in the contents of mineral elements; decrease in chlorophyll and soluble protein contents; induction of activities of antioxidant enzymes; growth inhibition at the highest concentration	[72]
			petioles	~13–70 g kg^−1^ dw		
			leaves	~3–45 g kg^−1^ dw		
*Plantago major*	0.01–100 mM in solution	7 weeks	leaves	8–105 mg kg^−1^ dw	Reduction in root and leaf biomass; decrease in chlorophyll fluorescence ^(3)^	[44]
*Raphanus* *sativus*	5–40 mg kg^−1^ in soil	60 days	roots	54–106 µg g^−1^ dw	Decrease in catalase and peroxidase activities; oxidative stress	[77]
			shoots	77–133 µg g^−1^ dw		
*Vicia faba*	5.86, 11.36 mM in solution	3 weeks	roots	~2100–3900 mg kg^−1^	Reduction in root and shoot biomass; inhibition of leaf development, chlorophyll accumulation and photosynthesis; induction of activities of antioxidant enzymes	[45]
			stems	~2800–6700 mg kg^−1^		
			leaves	~3200–7100 mg kg^−1^		
*Zea mays*	3 mM in solution	1 day	root a.p.	~10,230 µg g^−1^ dw	Decrease in the number of cells in the meristem row, the rate of cell production in the meristem and mitotic index in the cortical cells; increase in the length of the fully elongated cells	[84]
			root b.p.	~3640 µg g^−1^ dw		
		2 days	root a.p.	~18,860 µg g^−1^ dw		
			root b.p.	~3863 µg g^−1^ dw		

^(1)^ only at 40–160 mg kg^−1^ Sr in soil; ^(2)^ only at 500 mg kg^−1^ Sr in soil; ^(3)^ only at 0.01, 1, 100 mM Sr in solution; ^(4)^ only at 0.2–1 mmol L^−1^ Sr in solution. Abbreviations: root a.p.—root apical part (the first centimeter from the tip); root b.p.—root basal part (the third centimeter from the kernel).

**Table 2:** The effects of Sr on enzyme activities.

Enzyme/Process	Sr Content/ Concentration	Medium	Duration of Exposure	Plant Material	Enzyme Activity	Plant Species	Refs.
Ascorbate peroxidase EC 1.11.1.11 Reduction of hydrogen peroxide using ascorbate as an electron donor	0.3, 1.5, 3 mM	solution	21 days	plants	**⇑**	*Alstonia scholaris*	[46]
	0.3, 1.5, 3 mM	solution	30 days	plants	**⇑**	*Eichhornia crassipes*	[72]
	0.3, 1.5, 3 mM	solution	30 days	plants	**⇑**	*Monochoria hastata*	[72]
	10, 100 mg kg^−1^	soil	3 months	cuttings	**⇑**	*Populus alba*	[69]
	10 mg kg^−1^	soil	3 months	cuttings	**⇑**	*Populus russkii*	[69]
	100 mg kg^−1^				**⇓**		
	11.36 mM	solution	3 weeks	leaves	**⇑**	*Vicia faba*	[45]
Catalase EC 1.11.1.6 Conversion of toxic hydrogen peroxide into water and oxygen	0.3, 1.5, 3 mM	solution	21 days	plants	**⇑**	*Alstonia scholaris*	[46]
	1, 5 mmol kg^−1^	soil	until maturation	leaves	**⇑**	*Amaranthus* *mangostanus*	[81]
	2, 4, 6, 8, 10 mM	soil	7 days	plants	**⇑**	*Amaranthus caudatus*	[18]
	25, 100, 500 mg kg^−1^	soil	30 days	roots	**⇑**	*Avena sativa*	[66]
				leaves	**⇑**		
	10, 20, 40, 80, 160 mg kg^−1^	soil	60 days	leaves	**⇑**	*Cucumis sativus*	[83]
	0.3, 1.5, 3 mM	solution	30 days	plants	**⇑**	*Eichhornia crassipes*	[72]
	0.3, 1.5, 3 mM	solution	30 days	plants	**⇑**	*Monochoria hastata*	[72]
	5, 50 mg L^−1^	solution	7 days	plants	0	*Racomitrium* *japonicum*	[124]
	500 mg L^−1^				**⇑**		
	10 mM	solution		germinated seeds	0	*Triticum aestivum*	[125]
	100 mM				**⇑**		
	10 mM	solution		germinated seeds	0	*Triticum durum*	[125]
	100 mM				**⇑**		
	2.84, 5.86, 11.36 mM	solution	3 weeks	leaves	0	*Vicia faba*	[45]
Glutathione reductase EC 1.8.1.7 NADPH-dependent reduction in oxidized glutathione to the reduced form	0.3, 1.5, 3 mM	solution	21 days	plants	**⇑**	*Alstonia scholaris*	[46]
Glutathione S-transferase EC 2.5.1.18 Conjugation of electrophilic substrates to reduced glutathione	10 mM	solution		germinated seeds	0	*Triticum aestivum*	[125]
	100 mM				**⇑**		
	10 mM	solution		germinated seeds	0	*Triticum durum*	[125]
	100 mM				**⇑**		
Nitrate reductase EC 1.7.1.- Nitrate (NO_3_^−^) reduction to nitrite (NO_2_^−^)	2, 4, 6, 8, 10 mM	soil	7 days	plants	**⇓**	*Amaranthus caudatus*	[18]
Peroxidase EC 1.11.1.7 Reduction in toxic hydrogen peroxide using several reductants	0.3, 1.5, 3 mM	solution	21 days	plants	**⇑**	*Alstonia scholaris*	[46]
	1, 5 mmol kg^−1^	soil	until maturation	leaves	**⇓**	*Amaranthus* *mangostanus*	[81]
	2, 4, 6, 8, 10 mM	soil	7 days	plants	**⇑**	*Amaranthus caudatus*	[18]
	25, 100, 500 mg kg^−1^	soil	30 days	roots	**⇑**	*Avena sativa*	[66]
				leaves	**⇑** ^(1)^ 0 ^(2)^		
	20, 40, 80, 160 mg kg^−1^	soil	60 days	leaves	**⇑**	*Cucumis sativus*	[83]
	0.3, 1.5, 3 mM	solution	30 days	plants	**⇑**	*Eichhornia crassipes*	[72]
	0.3, 1.5, 3 mM	solution	30 days	plants	**⇑**	*Monochoria hastata*	[72]
	10, 100 mg kg^−1^	soil	3 months	cuttings	0	*Populus alba*	[69]
	10, 100 mg kg^−1^	soil	3 months	cuttings	0	*Populus russkii*	[69]
	5, 10, 20, 40 mg kg^−1^	soil	60 days	leaves	**⇓**	*Raphanus sativus*	[77]
	10 mM	solution		germinated seeds	0	*Triticum aestivum*	[125]
	100 mM				**⇑**		
	10 mM	solution		germinated seeds	0	*Triticum durum*	[125]
	100 mM				**⇑**		
	5.86, 11.36 mM	solution	3 weeks	leaves	0	*Vicia faba*	[45]
Phosphoenolpyruvate carboxylase EC 4.1.1.31 Carbon fixation	10, 20, 40 mM	solution	14, 21 days	leaves	**⇓**	*Brassica napus*	[74]
Ribulose-1,5-bisphosphate carboxylase/oxygenase EC 4.1.1.39 Carbon fixation	10, 20, 40 mM	solution	14, 21 days	leaves	**⇓**	*Brassica napus*	[74]
Superoxide dismutase EC 1.15.1.1 Dismutation of superoxide radicals into molecular oxygen and hydrogen peroxide	0.3, 1.5, 3 mM	solution	21 days	plants	**⇑**	*Alstonia scholaris*	[46]
	1, 5 mmol kg^−1^	soil	until maturation	leaves	**⇓**	*Amaranthus* *mangostanus*	[81]
	25, 100, 500 mg kg^−1^	soil	30 days	roots	**⇑**	*Avena sativa*	[66]
				leaves	**⇑**		
	20, 40, 80, 160 mg kg^−1^	soil	60 days	leaves	**⇑**	*Cucumis sativus*	[83]
	0.3, 1.5, 3 mM	solution	30 days	plants	**⇑**	*Eichhornia crassipes*	[72]
	0.3, 1.5, 3 mM	solution	30 days	plants	**⇑**	*Monochoria hastata*	[72]
	10, 100 mg kg^−1^	soil	3 months	cuttings	**⇑**	*Populus alba*	[69]
	10 mg kg^−1^	soil	3 months	cuttings	**⇑**	*Populus russkii*	[69]
	100 mg kg^−1^				**⇓**		
	10, 100 mM	solution		germinated seeds	**⇑**	*Triticum aestivum*	[125]
	10, 100 mM	solution		germinated seeds	**⇑**	*Triticum durum*	[125]
	2.84, 5.86, 11.36 mM	solution	3 weeks	leaves	0	*Vicia faba*	[45]

**⇓**—the activity of the enzyme decreased; **⇑**—the activity of the enzyme increased, 0—the activity of the enzyme did not change. The plants were grown in hydroponics (Sr concentration is given in mM, mg L^−1^) or in soil (mg kg^−1^ or mmol kg^−1^ Sr in soil). ^(1)^ only at 25 and 100 mg kg^−1^ Sr in soil in one of the varieties; ^(2)^ only at 500 mg kg^−1^ Sr in soil in both varieties.

**Table 3:** The effects of Sr on photosynthesis.

Plant Species	Sr Content/ Concentration	Chl	Car	*P*_n_	*F*_m_	*F*_v_/*F*_m_	qP	NPQ	*J*_max_ *ETR*	*g*_s_	*C*_i_	Refs.
*Amaranthus caudatus*	2, 4, 6, 8, 10 mM	**⇓**	**⇓**									[18]
*Amaranthus tricolor*	0.2, 0.6, 3, 6 mM					0	0	**⇑** ^(7)^				[27]
*Brassica rapa*	0.1–1 mM	**⇑** ^(1)^		0		0				0		[28]
	0.1–0.5 mM	**⇑**		0						0		[26]
	2–10 mM	**⇓**		0						0		
*Racomitrium* *japonicum*	50, 500 mg L^−1^	**⇓**				**⇓**		**⇑**				[124]
*Salix babylonica*	8 mM	**⇓**		**⇓**	**⇓**	**⇓**		**⇑**		**⇓**	**⇑**	[136]
*Solanum* *lycopersicum*	4 mM			**⇓**						**⇓**		[11]
*Vicia faba*	2.84, 5.86, 11.36 mM	**⇓**		**⇓** ^(2)^		**⇓**	**⇑** ^(4)^	**⇑** ^(5)^	**⇑** ^(6)^	**⇓**	**⇓** ^(3)^	[45]

**⇓**—decrease; **⇑**—increase, 0—no changes. Empty cells mean lack of data. Chlorophyll content (Chl), carotenoid content (Car), net photosynthetic rate (*P*_n_ (*A*), μmol CO_2_ m^−2^ s^−1^), maximal (*F*_m_) fluorescence yield, maximum quantum efficiency of PSII (*F*_v_/*F*_m_), photochemical quenching (qP), non-photochemical quenching (NPQ), electron transport rate (ETR, μmol e^−^ m^−2^ s^−1^) or maximum light-driven electron transport rate (*J*_max_, μmol m^−2^ s^−1^), stomatal conductance (*g*_s_, mol m^−2^ s^−1^), intracellular CO_2_ concentration (*C*_i_, mmol mol^−1^). ^(1)^ only at 0.2–1 mM Sr, ^(2)^ only at 5.86 and 11.36 mM Sr, ^(3)^ only at 11.36 mM Sr; ^(4)^ only at 5.86 mM Sr; ^(5)^ only at 2.84 and 5.86 mM Sr; ^(6)^ only at 2.84 mM Sr; ^(7)^ only at 3 mM Sr. The plants were grown in hydroponics (Sr concentration given in mM or mg L^−1^) or in soil (mg kg^−1^ Sr in soil).

**Table 4:** The effects of Sr on plant growth.

Plant Species	Sr Content/ Concentration	RL	SL	TW	RFW	RDW	SFW	SDW	LFW	LDW	RI	LA	NL	Refs.
*Allium cepa*	5–80 ppm	**⇓**												[146]
*Amaranthus caudatus*	2, 4, 6, 8 mM	**⇓**	**⇓**	**⇓**								**⇓**		[18]
*Amaranthus mangostanus*	0.5, 1, 5 mmol kg^−1^ Sr in soil					**⇓**		**⇓**						[81]
*Apium* *graveolens*	0.5, 1, 2.5 mM				0				**⇓**					[21]
*Arabidopsis* *halleri*	0.01 mM	0				0				0				[82]
	0.1 mM	**⇓**				0				0				
	1 mM	0				0				**⇑**				
	10 mM	0				0				**⇓**				
	100 mM	**⇓**				**⇓**				**⇓**				
*Arabidopsis* *thaliana*	10 mM	0		0										[143]
	20, 30, 40 mM	**⇓**		**⇓**										
*Brassica oleracea*	25 ppm			**⇓**		**⇓**		**⇓**		**⇓**				[71]
*Brassica rapa*	0.1 mM	0										0		[28]
	0.2–1 mM	**⇑**										0		
	0.2, 0.5 mM		**⇑**									**⇑**		[26]
	2, 5, 10 mM		**⇓**									**⇓**		
	100, 200 mg kg^−1^ Sr in soil	0		**⇓**	0	0	**⇓**	**⇓**				**⇓**	**⇓**	[40]
*Glycine max*	3 mM				**⇓**	**⇓**	**⇓**	**⇓**						[24]
*Cucumis sativus*	10, 20, 40, 80, 160 mg kg^−1^ Sr in soil	**⇓**		**⇓**		**⇓**		**⇓**				**⇓**		[83]
*Morus alba*	4 mM		**⇓**											[115]
*Plantago major*	0.01–10 mM	0				**⇓**				**⇓**				[44]
	100 mM	**⇓**				**⇓**				**⇓**				
*Vicia faba*	2.84, 5.86, 11.36 mM	**⇓**				**⇓**				**⇓**		**⇓**		[45]
*Zea mays*	3 mM										**⇓**			[84,100,104]
	10 mM	**⇓**				0		0						[13]

**⇓**—decrease; **⇑**—increase, 0—no changes. Empty cells mean lack of data. Root length (RL), shoot length (SL), total weight (TW), root fresh weight (RFW), root dry weight (RDW), shoot fresh weight (SFW), shoot dry weight (SDW), leaf fresh weight (LFW); leaf dry weight (LDW), root length increment (RI), leaf area (LA), number of leaves (NL).

## Data Availability

No new data were created or analyzed in this study. Data sharing is not applicable to this article.
